# Multi-UAV simultaneous target assignment and path planning based on deep reinforcement learning in dynamic multiple obstacles environments

**DOI:** 10.3389/fnbot.2023.1302898

**Published:** 2024-01-22

**Authors:** Xiaoran Kong, Yatong Zhou, Zhe Li, Shaohai Wang

**Affiliations:** ^1^School of Electronic and Information Engineering, HeBei University of Technology, Tianjin, China; ^2^Institute of Digital Economy Industry Research, Hebei University of Technology, Shijiazhuang, China; ^3^School of Electronic and Information Engineering, Nanjing University of Aeronautics and Astronautics, Nanjing, China

**Keywords:** multiple unmanned aerial vehicles, target assignment, path planning, deep reinforcement learning, partially observable Markov decision process

## Abstract

Target assignment and path planning are crucial for the cooperativity of multiple unmanned aerial vehicles (UAV) systems. However, it is a challenge considering the dynamics of environments and the partial observability of UAVs. In this article, the problem of multi-UAV target assignment and path planning is formulated as a partially observable Markov decision process (POMDP), and a novel deep reinforcement learning (DRL)-based algorithm is proposed to address it. Specifically, a target assignment network is introduced into the twin-delayed deep deterministic policy gradient (TD3) algorithm to solve the target assignment problem and path planning problem simultaneously. The target assignment network executes target assignment for each step of UAVs, while the TD3 guides UAVs to plan paths for this step based on the assignment result and provides training labels for the optimization of the target assignment network. Experimental results demonstrate that the proposed approach can ensure an optimal complete target allocation and achieve a collision-free path for each UAV in three-dimensional (3D) dynamic multiple-obstacle environments, and present a superior performance in target completion and a better adaptability to complex environments compared with existing methods.

## 1 Introduction

Recently, unmanned aerial vehicles (UAV) have been widely applied to a variety of fields due to their advantages of high flexibility, low operating cost, and ease of deployment. In the military field, UAVs have become an important part of modern warfare and can be used for missions such as reconnaissance (Qin et al., 2021), strikes (Chamola et al., 2021), and surveillance (Liu et al., 2021), reducing casualties and enhancing combat efficiency. In the field of agriculture, UAVs have good applications in plant protection (Xu et al., 2019; Chen et al., 2021), agricultural monitoring (Zhang et al., 2021), and so on, improving the efficiency and precision of agricultural operations. In the field of environmental protection, UAVs are extensively employed in environmental monitoring (Yang et al., 2022), pollution source tracking (Liu et al., 2023), nature reserve inspection (Su et al., 2018), and other tasks, effectively supporting environmental protection work. In addition, for search and rescue tasks (Fei et al., 2022; Lyu et al., 2023), UAVs can quickly obtain disaster information through airborne sensors to provide efficient and timely assistance for subsequent rescue. However, it is difficult to apply lone single UAVs to complex and diverse missions due to their limited functionality and payload. Cooperation between multiple UAVs (Song et al., 2023) has greatly expanded the ability and scope of task execution, and has gradually replaced the single UAV as the nontrivial technology for various complex tasks. The key to solving the multi-UAV cooperative problems (Wang T. et al., 2020; Xing et al., 2022; Wang et al., 2023) is target assignment and path planning for UAVs, which is the guarantee of task completion.

The above problem consists of two fundamental sub-problems. Target assignment (Gerkey and Matarić, 2004) means assigning one UAV for each target to maximize the overall efficiency or minimize the total costs. It has many effective solutions such as the Genetic algorithm (GA) (Tian et al., 2018) and the Hungarian algorithm (Kuhn, 1955). Lee et al. (2003) introduced greedy eugenics to GA to improve the performance of GA in weapon-target assignment problems. Aiming at the multi-task allocation problem, Samiei et al. (2019) proposed a novel cluster-based Hungarian algorithm. Path planning (Aggarwal and Kumar, 2020) refers to each drone planning an optimal path from its initial location to its designated target with the collision-free constraint. It has been studied extensively, and A* (Grenouilleau et al., 2019), rapidly-exploring random tree algorithm (RRT) (Li et al., 2022) and particle swarm optimization (PSO) (Fernandes et al., 2022) are classical methods. Fan et al. (2023) incorporated the artificial potential field method into RRT to reduce the cost of path planning. He W. et al. (2021) proposed a novel hybrid algorithm for UAV path planning by combining PSO with the symbiotic organism search. While most previous works tackle the problem in static environments, and a common feature of these solutions is that they rely on global information of the task environment for explicit planning, which may lead to unexpected failure in the face of uncertain circumstances or unpredictable obstacles.

Therefore, some studies resort to learning-based approaches such as deep learning (DL) (Kouris and Bouganis, 2018; Mansouri et al., 2020; Pan et al., 2021). Pan et al. (2021) combined DL and GA to plan the path for UAV data collection. The proposed method collected various paths and states in different task environments by GA, and used them to train the neural network of DL, which can give an optimal path in familiar scenarios with real-time requirements. Kouris and Bouganis (2018) proposed a self-supervised CNN-based approach for indoor UAV navigation. This method used an indoor-flight dataset to train the CNN and utilized the CNN to predict collision distance based on an on-board camera. However, Deep learning-based approaches require labels for learning and they are infeasible when the environment is highly variable.

Unlike DL methods, reinforcement learning (RL) (Thrun and Littman, 2000; Busoniu et al., 2008; Zhang et al., 2016) can optimize strategies directly through trial-and-error iteration interacting with the environment without prior knowledge, which is adaptable to dynamic environments. Moreover, deep reinforcement learning (DRL) (Mnih et al., 2015) combines DL and RL to implement end-to-end learning. It makes RL no longer limited to low-dimensional space and greatly expands the scope of application of RL (Wang C. et al., 2020; Chane-Sane et al., 2021; He L. et al., 2021; Kiran et al., 2021; Luo et al., 2021; Wu et al., 2021; Yan et al., 2022; Yue et al., 2023; Zhao et al., 2023). Wu et al. (2021) introduced a curiosity-driven method into DRL to improve training efficiency and performance in autonomous driving tasks. Yan et al. (2022) proposed a simplified, unified, and applicable DRL method for vehicular systems. Chane-Sane et al. (2021) designed a new RL method with imagined possible subgoals to facilitate learning of complex tasks such as challenging navigation and vision-based robotic manipulation. Luo et al. (2021) designed a DRL-based method to generate solutions for the missile-target assignment problem autonomously. He L. et al. (2021) presented an autonomous path planning method based on DRL for quadrotors in unknown environments. Wang C. et al. (2020) proposed DRL algorithm with nonexpert helpers to address the autonomous navigation problem for UAVs in large-scale complex environments.

DRL is suitable to solve the target assignment problem and path planning problem of UAVs, but there are still some challenges when multiple UAVs perform tasks in dynamic environments. The first challenge is inefficient target assignment. Typically, UAVs execute target assignment first and then perform path planning based on the result of the target assignment. However, the dynamism and uncertainty of the environment always lead to an inaccurate assignment result, which directly affects the subsequent path planning. In this respect, UAVs need to perform autonomous target assignment and path planning simultaneously. There are only a few scholars who have studied this field. Qie et al. (2019) constructed the multiple UAVs target assignment and path planning problem as a multi-agent system and used the multi-agent deep deterministic policy gradient (MADDPG) (Lowe et al., 2017) framework to train the system to solve two problems simultaneously. They traverse all targets and select the agent closest to each target after each step of the agent, which often results in an incomplete assignment of targets when two agents are at the same and shortest distance from one target. Han et al. (2020) proposed a navigation policy for multiple robots in a dynamic environment based on the Proximal Policy Optimization (PPO) (Schulman et al., 2017) algorithm. The target assignment scheme was proposed depending on the distance between robots and targets. However, this assignment method does not take into account the obstacles in the task environment, which is vulnerable to leads to inaccurate allocation in a multi-obstacle environment similar to the real world. The second challenge is that UAVs' onboard sensors have limited detection range. The real-time decision-making of UAVs depends on observation returned by sensors, especially in dynamic and uncertain environments. If the detection range of sensors is limited, the current state cannot fully represent the global environmental information, which greatly increases the difficulty of autonomous flight.

To overcome these challenges, this article models the multi-UAV target assignment and path planning problem as a partially observable Markov decision process (POMDP) (Spaan, 2012) and designs a simultaneous target assignment and path planning method based on DRL to settle it. Among the DRL-based methods, the twin-delayed deep deterministic policy gradient (TD3) (Fujimoto et al., 2018) is a state-of-the-art (SOTA) DRL algorithm and has been widely used in training the policy of UAVs. It significantly improves the learning speed and performance of deep deterministic policy gradient (DDPG) (Lillicrap et al., 2015) algorithm by reducing the overestimation of DDPG. Zhang et al. (2022) introduced the spatial change information of environment to the TD3, and used it to guide a UAV to complete navigation tasks in complex environments with multiple obstacles. Hong et al. (2021) proposed an advanced TD3 model to perform energy-efficient path planning at the edge-level drone. In this regard, a more effective DRL algorithm based on TD3 is proposed to solve the POMDP in this article.

The main contributions of this article can be summarized as follows:

- A DRL framework for multi-UAV target assignment and path planning is developed in 3D dynamic multiple obstacles environments, where the target assignment and path planning problem is modeled as a POMDP.
- A simultaneous target assignment and path planning method taking into account UAVs, targets, and moving obstacles is proposed, which can achieve an optimal target assignment and complete collision-free path planning for each UAV simultaneously.
- A 3D stochastic complex simulation environment is built to train an algorithm, and the experimental results validate the effectiveness of the proposed method.

The remainder of this article is organized as follows: The background is presented in Section 2, Section 3 introduces the formulation of the multi-UAV problem. In Section 4, a detailed introduction to our method is provided. Section 5 presents the simulation experiments and results. Finally, the conclusion of this paper and future work are summarized in Section 6.

## 2 Background

This section gives a brief introduction to the multi-UAV target assignment and path planning problem in this article first, followed by the multi-UAV problem formulated as a POMDP in 3D dynamic environments.

### 2.1 Multi-UAV target assignment and path planning problem

**Figure 1:**
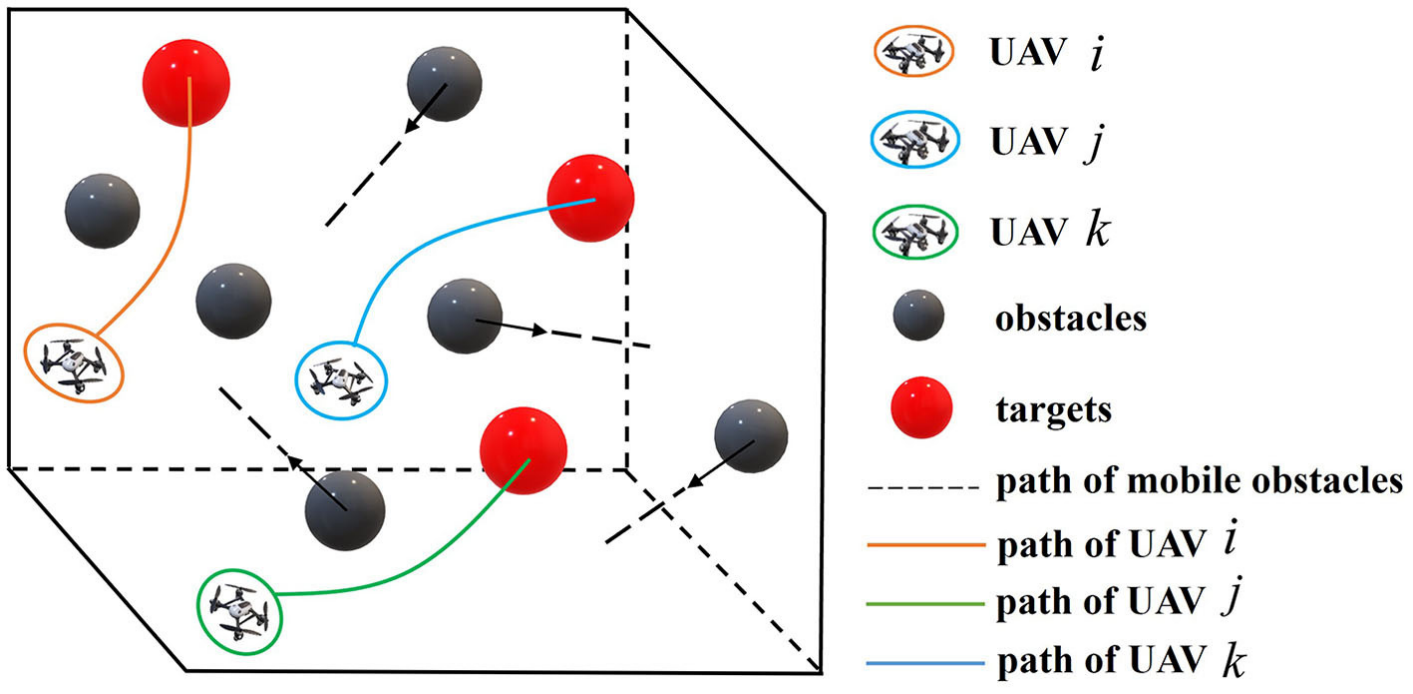
Schematic diagram of multi-UAV target assignment and path planning.

The multiple UAVs target assignment and path planning scenario of this paper is shown in Figure 1:

(1) A series of UAVs are commanded to fly across a 3D mission area until they reach the targets distributed in different locations.

(2) The mission area is scattered with some static or irregularly moving obstacles.

(3) UAVs are required to avoid collision with each other and obstacles.

(4) UAVs are isomorphic and targets are identical.

The object of multi-UAV target assignment and path planning is to minimize the total flight path length of all UAVs [Equation (1)] under the constraints of target completely assignment and collision-free:


(1) $$\min\left(\sum_{i}^{N_{U}}d_{i}\right)$$

(2) $$\left\{ \begin{array}{l} \cup_{i=1}^{N_{U}}T_{i}=T,\;i\in \{1,\ldots ,N_{T}\},\\ T_{i}\neq T_{j},\;i\neq j. \end{array}\right.$$

(3) $$\left\{ \begin{array}{l} \left\|U_{i}^{t},U_{j}^{t}\right\|>2r_{u},\;i,j=1,2,\ldots ,N_{U},\\ \left\|U_{i}^{t},O_{k}^{t}\right\|>r_{u}+r_{o},\;i=1,2,\ldots ,N_{U},\;k=1,2,\ldots ,M. \end{array}\right.$$

where *T*_*i*_∈**T**, *i*∈{1, …, *N*_*T*_} denotes the targets, *U*_*i*_, *i* = 1, 2, …, *N*_*U*_ denotes the UAVs and *O*_*k*_, *k* = 1, 2, …, *M* denotes the obstacles. $U_{i}^{t},\;O_{k}^{t}$ represents the positions of UAV *i* and obstacle *k* at time *t*, respectively. *d*_*i*_ is the flight length of UAV *i*, *r*_*u*_ and *r*_*o*_ are the radius of UAVs and obstacles. Equation (2) denotes the target complete assignment constraint, which means each target is only assigned to one UAV. Equation (3) defines the collision-free constraint, where the first one means any two UAVs cannot collide at all times, while the second defines each UAV's path is collision-free with obstacles.

### 2.2 Modeling multiple UAVs problem as an POMDP

The multi-UAV problem can be modeled as POMDP, which is composed of a tuple 〈**N, S, O**, **A, P, R**〉. In this tuple, **N** = {1, 2, ⋯ , *N*} represents the collection of *N* UAVs, **S** is the state space of UAVs, **O** = {*o*_1_, *o*_2_, ⋯ , *o*_*N*_} is the observation of all UAVs, where *o*_*i*_ represents the observation of UAV *i*. When the environment is partially observable, at time *t*, each UAV only obtains its own local observation *o*_*t, i*_∈*o*_*i*_. **A** = {*a*_1_, *a*_2_, ⋯ , *a*_*N*_} is the action collection of UAVs, where *a*_*i*_ is the action taken by UAV *i*; **P**:**S**×**A**×***S*′**∈[0, 1] denotes the probability that state transfers from **S** to ***S*′** after performing action **A**; **R** = {*R*_1_, *R*_2_, ⋯ , *R*_*N*_} is the reward collection of UAVs, where *R*_*i*_ denotes the reward of UAV *i* received from the environment.

**Figure 2:**
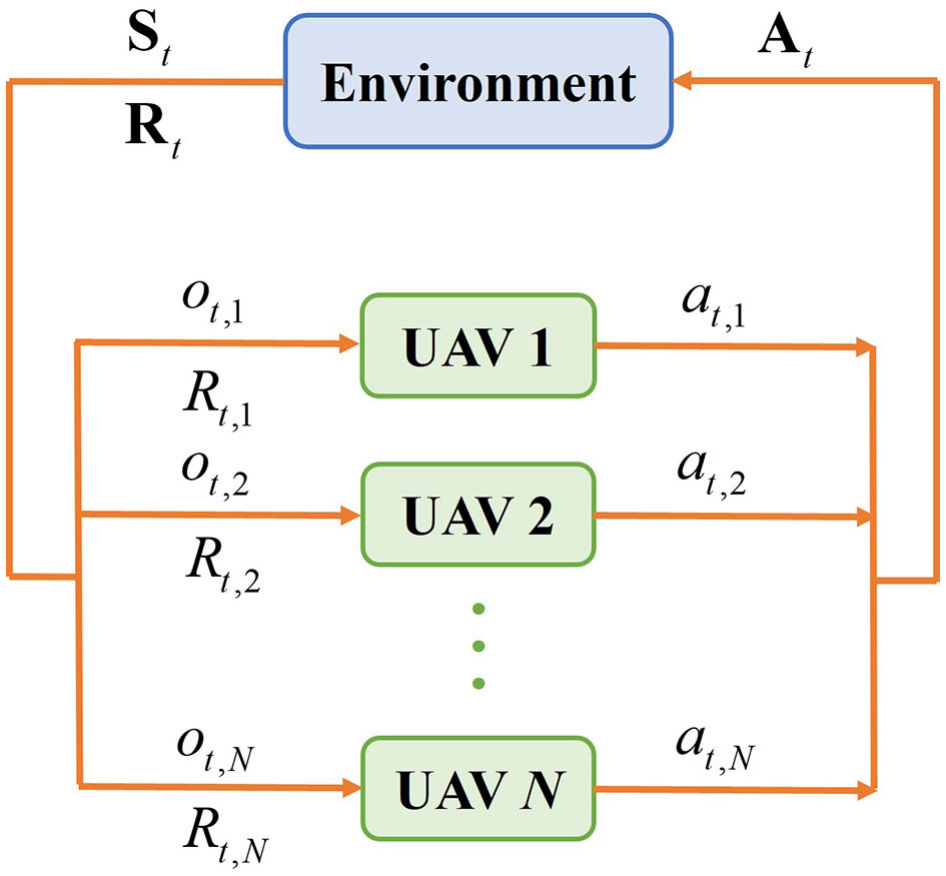
Multi-UAV reinforcement learning process in partially observable environment.

The multi-UAV reinforcement learning process in a partially observable environment is shown in Figure 2. At each epoch *t*, UAV *i* selects its optimal action *a*_*t, i*_ based on the policy π to maximize the joint cumulative reward of all UAVs, and π(*a*|*s*) = *P*[*A*_*t*_ = *a*|*S*_*t*_ = *s*] represents the probability of action *a* under state *s*. Then the joint action **A**_*t*_ = {*a*_*t*, 1_, *a*_*t*, 2_, …, *a*_*t, N*_} of UAVs is executed to control the movement of UAVs, the joint state is changed to **S**_*t*+1_ and the reward received by the UAV *i* is *R*_*t, i*_. The cumulative reward of UAV i is defined as Equation (4),


(4) $$\begin{array}{l} {J_{i}}^{\pi }=\overset{T}{\underset{t=0}{\sum }}\gamma^{t}R_{t,i} \end{array}$$

where γ∈[0, 1] is the discount factor that balances the current rewards and the future rewards.

## 3 Problem formulation

**Figure 3:**
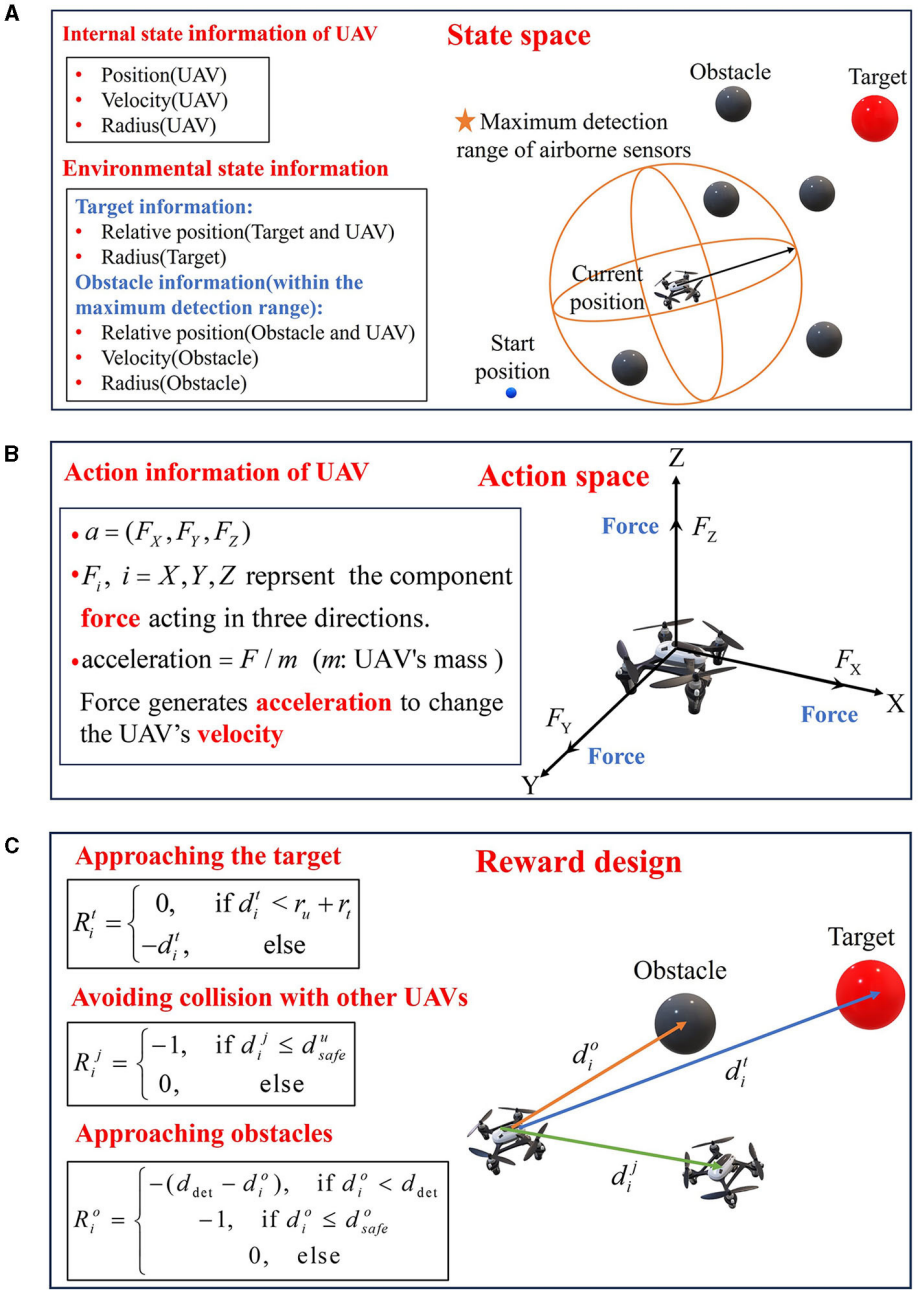
Problem formulation. **(A)** State space. **(B)** Action space. **(C)** Reward design.

UAVs use onboard sensors to acquire their internal state information and environmental state information, execute actions according to the DRL model, and obtain the corresponding reward. Figure 3 describes the problem formulation.

### 3.1 State space

The state space consists of the internal state of the UAV and the environmental information within the max detection distance *d*_det_ of onboard sensors. The state space of UAV *i* can be defined as *s*_*i*_ = (*s*_*ui*_, *o*_*i*_). *s*_*ui*_ = (*p*_*i*_, *v*_*i*_, *r*_*i*_) is the internal state of UAV *i*, which is composed of the position *p*_*i*_ = (*x*_*i*_, *y*_*i*_, *z*_*i*_), the velocity *v*_*i*_ and the radius *r*_*i*_ of UAV *i*. *o*_*i*_ = (*s*_*T*_, *s*_*U*_, *s*_*O*_) is the environmental information observed by UAV *i*, where *s*_*T*_ = (*p*_*t*_, *r*_*t*_) is the relative position *p*_*t*_ = (*x*_*t*_−*x*_*i*_, *y*_*t*_−*y*_*i*_, *z*_*t*_−*z*_*i*_) to the target with the radius *r*_*t*_, *s*_*U*_ = (*p*_*u*_, *v*_*u*_, *r*_*u*_) is the relative position *p*_*u*_ = (*x*_*u*_−*x*_*i*_, *y*_*u*_−*y*_*i*_, *z*_*u*_−*z*_*i*_) to other UAVs with the velocity *v*_*u*_ and the radius *r*_*u*_. *s*_*O*_ represents the state of obstacles. If obstacles are within the max detection range, *s*_*O*_ = (*p*_*o*_, *v*_*o*_, *r*_*o*_) is the relative position *p*_*o*_ = (*x*_*o*_−*x*_*i*_, *y*_*o*_−*y*_*i*_, *z*_*o*_−*z*_*i*_), the velocity *v*_*o*_ and the radius *r*_*o*_ of the obstacles, otherwise, *s*_*O*_ = (±*d*_det_, ±*d*_det_, ±*d*_det_, 0, 0).

### 3.2 Action space

In this paper, the action space of UAV *i* is defined as *a*_*i*_ = (*F*_*i*_*X*__, *F*_*i*_*Y*__, *F*_*i*_*Z*__) as shown in Figure 3B, where the *F*_*i*_*X*__, *F*_*i*_*Y*__, *F*_*i*_*Z*__ represent the component forces applied to UAV *i* in *X*, *Y*, and *Z* three directions, respectively. The force produces an acceleration to change the velocity of the UAV.

### 3.3 Reward function

In this paper, the goal of the reward function is to guide UAVs to fly to the assigned target without any collision. In order to address the problem of underperforming training efficiency caused by sparse rewards, the reward function in this article uses a combination of guided rewards and sparse rewards. In the process of interacting with the environment, if a UAV reaches the target, collides with other UAVs, or hits an obstacle, a sparse reward is applied; when none of these three situations occurs, a guided reward is applied.

(1) Approaching the target

This reward function is to guide the UAV to head for the target and reach the target. When a UAV moves away from the target, it will receive a larger penalty related to the distance between the UAV and the target, and a reward of value 0 will be given to the UAV when it arrives at the target. Consequently, the reward for UAV *i* approaching the target can be defined as Equation (5),


(5) $$R_{i}^{t}=\left\{ \begin{array}{l} 0,\text{ if }d_{i}^{t}<r_{u}+r_{t}\\ -d_{i}^{t},\text{ else} \end{array}\right.$$

where $d_{i}^{t}$ denotes the distance between UAV *i* and the target, *r*_*u*_ is the radius of UAVs, *r*_*t*_ is the radius of targets.

(2) Avoiding collision with other UAVs

This reward is to avoid collision with other UAVs in the process of approaching the target. When the distance between UAV *i* and UAV *j* is shorter than their minimum safe distance, a collision will occur and the penalty value is set as Equation (6),


(6) $$R_{i}^{j}=\left\{ \begin{array}{l} -1,\text{ if }d_{i}^{j}\leq d_{safe}^{u}\\ 0,\text{ else} \end{array}\right.$$

where $d_{i}^{j}$ represents the distance between UAV *i* and UAV *j*, $d_{safe}^{u}=2r_{u}$ is the minimum safe distance between UAVs.

(3) Avoiding obstacles

The aim of this reward function is to keep UAVs away from obstacles. If the obstacle appears within the detection range *d*_det_, the UAV will obtain a punishment, and the closer the UAV gets to the obstacle, the greater the penalty. When the distance between the UAV and the obstacle is less than their minimum safe distance, a penalty of −1 will be given to the UAV.


(7) $$R_{i}^{o}=\left\{ \begin{array}{l} -(d_{\det}-d_{i}^{o}),\text{ if }d_{i}^{o}<d_{\det}\\ \;-1,\text{ if }d_{i}^{o}\leq d_{safe}^{o}\\ \;0,\text{ else} \end{array}\right.$$

In Equation (7), $d_{i}^{o}$ is the distance between UAV *i* and the nearest obstacle within the detection range, $d_{safe}^{o}=r_{u}+r_{o}$ is the minimum safe distance between UAV and obstacles, *r*_*o*_ is the radius of obstacles.

In conclusion, the reward function received by UAV *i* can be summarized as Equation (8),


(8) $$\begin{array}{l} R_{i}=R_{i}^{t}+R_{i}^{j}+R_{i}^{o} \end{array}$$

As can be seen from the reward function designed in this article, the guided reward functions are all negative. It means that each additional step taken by the UAV will have a negative value as a step penalty before reaching the target. Therefore, the reward value in this article can reflect the length of the flight path. A longer flight path corresponds to a smaller reward value.

## 4 Algorithm

In this section, the proposed algorithm, TANet-TD3, is illustrated in detail.

### 4.1 TD3 algorithm

This paper uses the TD3 as a basic algorithm to address the multi-UAV target assignment and path planning problem. As an improvement of the DDPG algorithm, TD3 also uses an Actor-Critic structure, but it introduces three technologies to prevent the overestimation problem of DDPG:

(1) Clipped double-Q learning.

**Figure 4:**
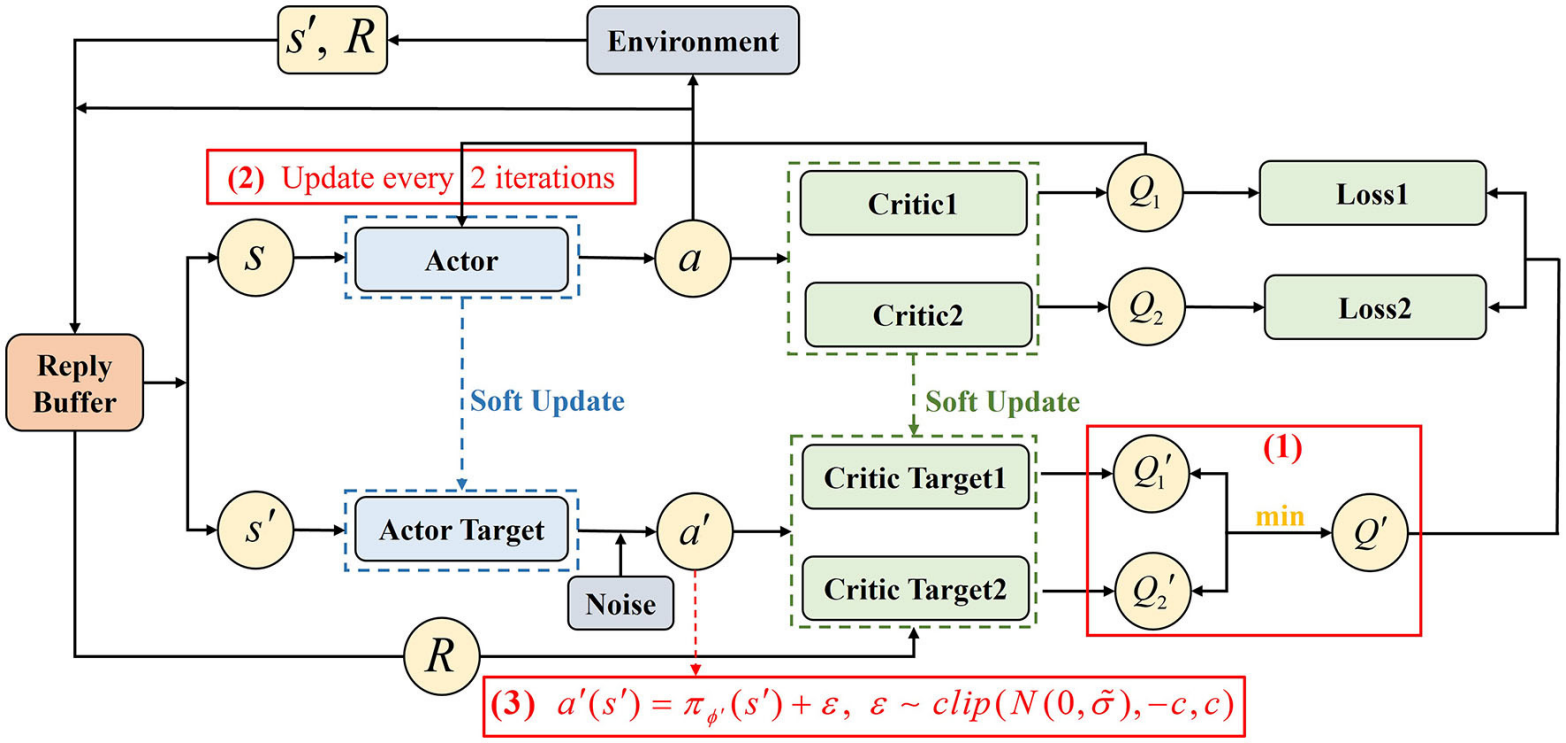
TD3 algorithm architecture.

TD3 has two Critic networks *Q*_θ_*n*__ parameterized by θ_*n*_, *n* = 1, 2 and two Critic target networks $Q_{{\theta^{\prime }}_{n}}$ parameterized by ${\theta^{\prime }}_{n},n=1,2$. The smaller one of two target *Q*-values is used to calculate the target value function *y* [Equation (9)] to alleviate the overestimation problem of the value function, as shown in (1) of Figure 4.


(9) $$\begin{array}{l} y=R\left(s,a\right)+\gamma \underset{n=1,2}{\min}Q_{{\theta^{\prime }}_{n}}\left(s^{\prime },a^{\prime }\right) \end{array}$$

Therefore, the two Critic networks are updated by minimizing the loss function as Equation (10),


(10) $$\begin{array}{l} \theta_{n}\leftarrow \arg\min_{\theta_{n}}N^{-1}\sum {\left(Q_{\theta_{n}}\left(s,a\right)-y\right)}^{2},\text{n}=1,2 \end{array}$$

(2) Delayed policy update.

TD3 updates the policy after getting an accurate estimation of the value function to ensure more stable training of the Actor-network. Usually updating the Actor once when the Critic is updated twice, as shown in (2) of Figure 4.

(3) Target policy smoothing.

The policy in DDPG is susceptible to influence by the function approximation error. TD3 adds the clipped noise into the target policy to make the value estimate more accurate, as shown in (3) of Figure 4.


(11) $$\begin{array}{l} a^{\prime }\left(s^{\prime }\right)=\pi_{\phi^{\prime }}\left(s^{\prime }\right)+\varepsilon ,\;\varepsilon \sim clip\left(N\left(0,\tilde{\sigma }\right),-c,c\right) \end{array}$$

In Equation (11), *s*′ and *a*′ represent the state and action at the next time, respectively. $\pi_{\phi^{\prime }}$ represents the Actor target network with the parameter ϕ′ and ε denotes the clipped noise.

Each UAV executes action *a* to transform the state *s* to next state *s*′, and obtains a reward *R* from the environment. The data (*s, a, R, s*′) is stored in the replay buffer *D* as a tuple. Sample a minibatch transition randomly from *D*, and input the *s*′ into the Actor target network $\pi_{\phi^{\prime }}$ to get the next action *a*′. Then, input the (*s*′, *a*′) into the two Critic target networks $Q_{{\theta^{\prime }}_{1}},\;Q_{{\theta^{\prime }}_{2}}$ to calculate the *Q*-values and select the smaller one to calculate the target value *y*. In the meantime, input (*s, a*) into the two Critic network *Q*_θ_1__, *Q*_θ_2__ and calculate the MSE with *y* to update the parameters θ_1_, θ_2_ of two Critic networks. After that, input the *Q*-value acquired from Critic network *Q*_θ_1__ into the Actor-network π_ϕ_, and update its parameter ϕ in the direction of increasing the *Q*-value as Equation (12),


(12) $$\begin{array}{l} \nabla_{\phi }J\left(\phi \right)=N^{-1}\sum \nabla_{a}Q_{\theta_{1}}\left(s,a\right){|}_{a=\pi_{\phi }\left(s\right)}\nabla_{\phi }\pi_{\phi }\left(s\right) \end{array}$$

Finally, the target Actor network' parameter ϕ′ and the two target Critic networks' parameters ${\theta^{\prime }}_{1},\;{\theta^{\prime }}_{2}$ are updated by soft update as follows Equation (13) and Equation (14),


(13) $$\begin{array}{l} {\theta_{n}}^{\prime }=\tau \theta_{n}+\left(1-\tau \right){\theta_{n}}^{\prime },\;n=1,\;2, \end{array}$$

(14) $$\begin{array}{l} \phi^{\prime }=\tau \phi +\left(1-\tau \right)\phi^{\prime }. \end{array}$$

### 4.2 TANet-TD3

#### 4.2.1 Framework of the TANet-TD3

**Figure 5:**
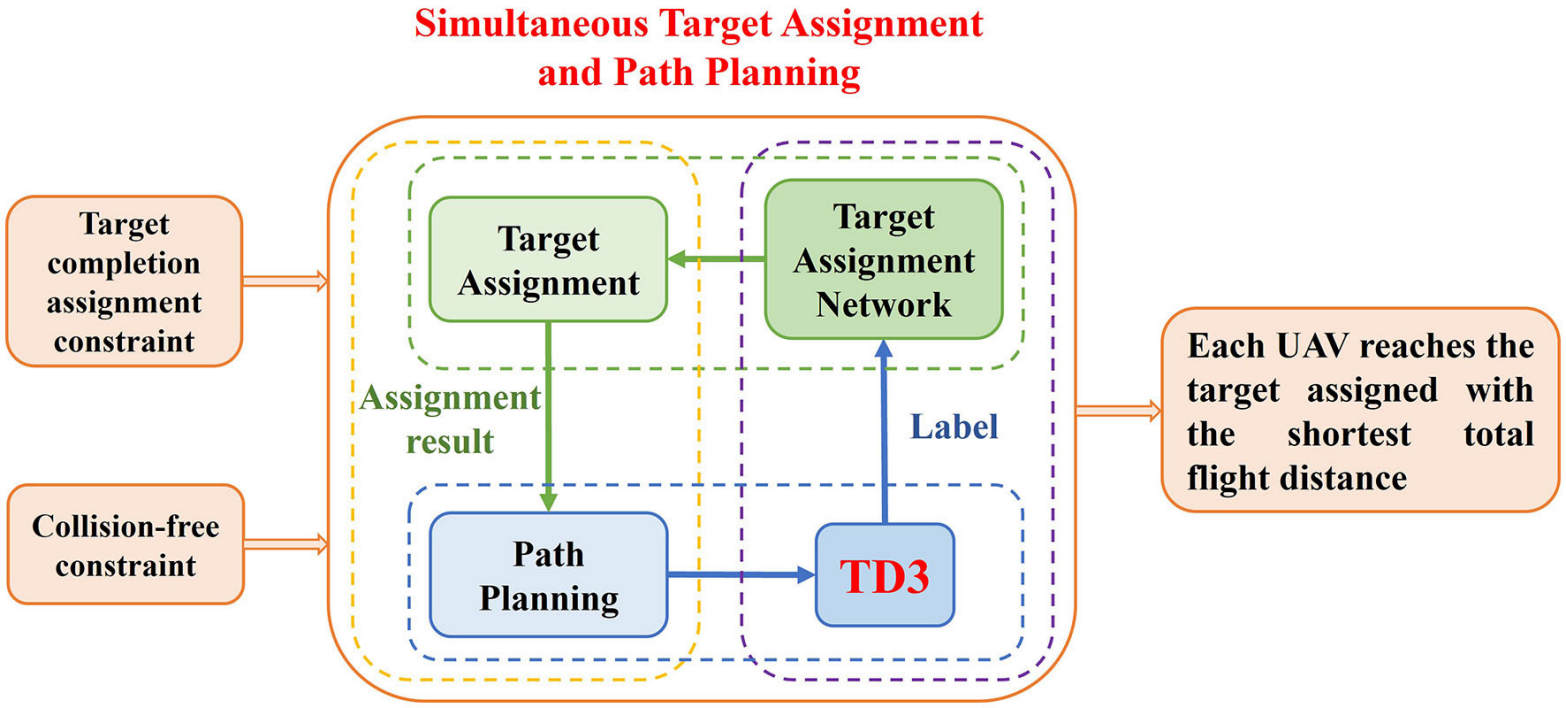
The framework of TANet-TD3.

This paper proposed the twin-delayed deep deterministic policy gradient algorithm with target assignment network (TANet-TD3), different from the existing methods that assign targets for the whole task first and then planning the path according to the assignment results, TANet-TD3 can solve the multiple UAVs target assignment and path planning simultaneously in dynamic multi-obstacle environments. The framework of the TANet-TD3 is shown in Figure 5, it can be seen that the object of the task is to minimize the total flight path length of all UAVs with the complete target assignment constraint and collision-free constraint. TANet-TD3 introduces a target assignment network into the framework of TD3 to solve the two problems simultaneously. Among the overall process, the target assignment network provides the optimal complete assignment of targets for each step of UAVs (the green dashed box), and then the TD3 algorithm guides each UAV plan a feasible path for this step (the blue dashed box) according to the assigned result (the yellow dashed box). In the meantime, the training labels of assignment network are obtained from the process of path planning driven by TD3 algorithm (the purple dashed box). This method not only takes into account the distance between UAVs and targets but also considers the dynamic obstacles in task environments, so it can generate an optimal assignment and path.

#### 4.2.2 Framework of target assignment

**Figure 6:**
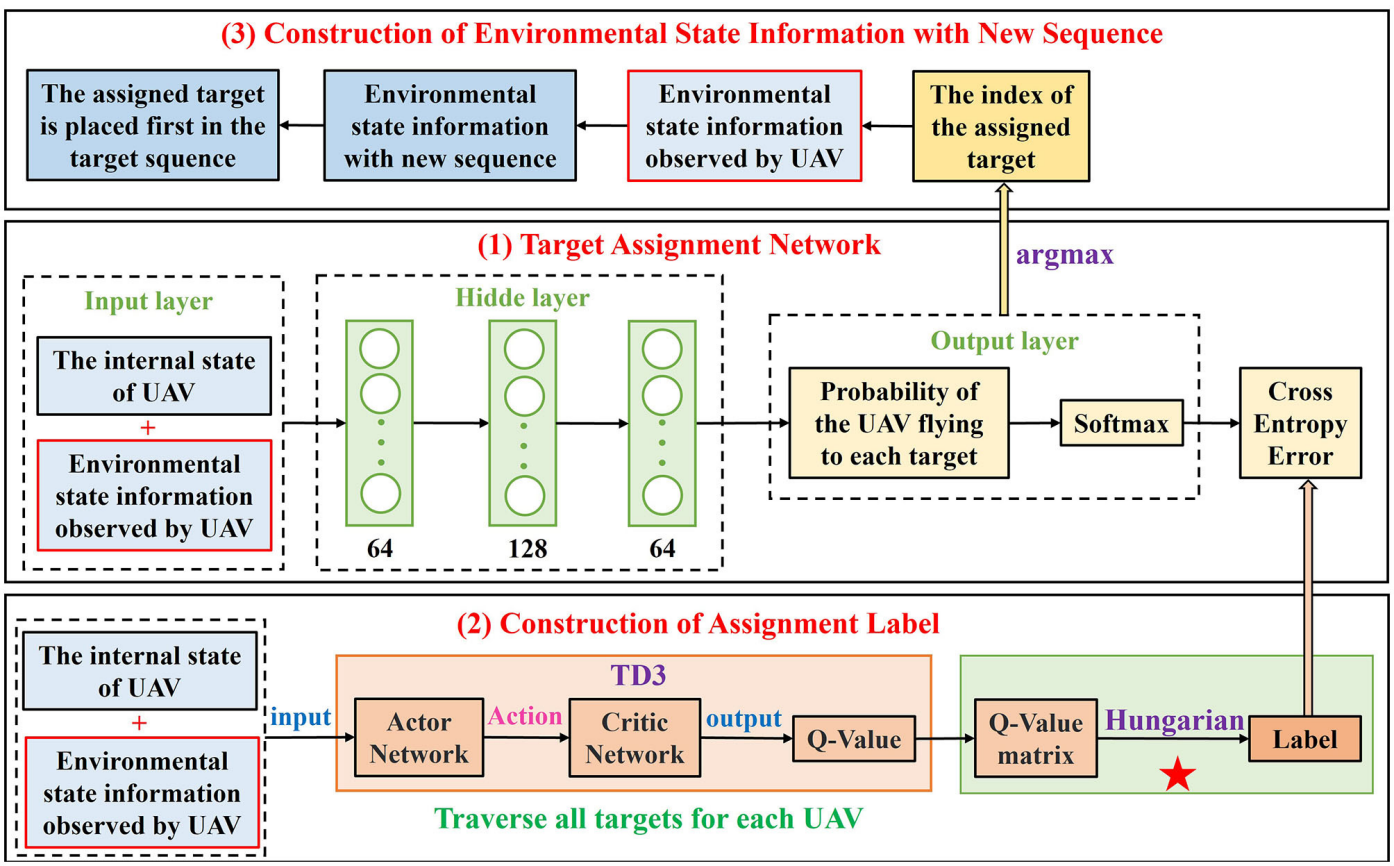
The framework of target assignment.

Figure 6 illustrates the overall framework of target assignment. It is composed of three parts, including the target assignment network, construction of the assignment label, and construction of the environmental state information with new sequence.

(1) Target assignment network

The network structure of target assignment network is designed as the middle section of Figure 6, it consists of a (7+4(*N*_*U*_−1)+4*N*_*T*_+7*N*_*O*_) × 64 × 128 × 64 × *N*_*T*_ fully-connected neural network layers, where (7+4(*N*_*U*_−1)+4*N*_*T*_+7*N*_*O*_) represents the state information *s*_*i*_ = (*s*_*ui*_, *o*_*i*_) of each UAV under the scenario of *N*_*U*_ UAVs, *N*_*T*_ targets and *N*_*O*_ obstacles within the detection range. For UAV *i*, after four FCs, the target assignment network maps the state information *s*_*i*_ = (*s*_*ui*_, *o*_*i*_) to the probability (*P*_*i*_*T*__1__, *P*_*i*_*T*__2__, ⋯ , *P*_*i*_*T*__*N*_*T*___) of UAV *i* flying to targets (*T*_1_, *T*_2_, ⋯ , *T*_*N*_*T*__). The probability is first normalized by the Softmax function [Equation (15)],


(15) $$\begin{array}{l} p_{ij}=\frac{p_{iT_{j}}}{\sum_{j}^{N_{T}}p_{iT_{j}}} \end{array}$$

and then the Cross-Entropy calculation is performed with the assigned labels to update the assignment network [Equation (16)],


(16) $$\begin{array}{l} H\left(L,P\right)=-\sum_{j}^{N_{T}}l_{j}\log p_{ij} \end{array}$$

(2) Construction of the assignment label

From the bottom section of the Figure 6, it can be seen that the training labels of the assignment network are provided by TD3 framework. The task objective is to achieve a complete assignment and minimize the total flight path, but it is not accurate to only consider the distance between UAVs and targets to make decisions in random and dynamic environments. As mentioned in Section 2.2, a multi-UAV problem means to maximize the joint cumulative reward of all UAVs in DRL, that is, each UAV will choose the action that maximized the *Q*-value based on its current state. Compared with selecting the target only according to distance, this method determines the assigned target according to the *Q*-value comprehensively taking into account UAVs, targets, and obstacles, even if obstacles are moving, so the targets can get an optimal assignment.

For UAV *i*, a 1 × *N*_*T*_ *Q*-value list (*Q*_*i*1_, *Q*_*i*2_, …, *Q*_*i*_*N*__*T*__) can be obtained for each step from the initial position by considering each target *T*_*j*_, *j* = 1, 2, …, *N*_*T*_ as the destination the UAV *i* will eventually reach, and for *N*_*U*_ UAVs, a *N*_*U*_×*N*_*T*_ value matrix is formed as Equation (17) by traversing all targets,


(17) $$\left(\begin{array}{cccc} Q_{11}, & Q_{12}, & \ldots , & Q_{1N_{T}}\\ Q_{21}, & Q_{22}, & \ldots , & Q_{2N_{T}}\\ \ldots , & \ldots , & \ldots , & \ldots ,\\ Q_{N_{U}1}, & Q_{N_{U}2}, & \ldots , & Q_{N_{U}N_{T}} \end{array}\right)$$

In order to ensure the constraints of complete target assignment, among many methods, the Hungarian algorithm has fast solution speed and stable solution quality, and with the aid of the independent 0 element theorem, it can obtain the exact solution of the problem by making elementary changes for the matrix with finite steps. Therefore, the Hungarian algorithm is introduced to achieve a complete allocation for targets in this article. After the Hungarian transformation, the *Q*-value matrix can be transformed into a permutation matrix with only 0 and 1 elements such as in Equation (18)


(18) $$\begin{array}{c} \left(\begin{array}{cccc} Q_{11}, & Q_{12}, & \ldots , & Q_{1N_{T}}\\ Q_{21}, & Q_{22}, & \ldots , & Q_{2N_{T}}\\ \ldots , & \ldots , & \ldots , & \ldots ,\\ Q_{N_{U}1}, & Q_{N_{U}2}, & \ldots , & Q_{N_{U}N_{T}} \end{array}\right)\overset{\text{Hungarian}}{\to }i{\overset{j}{\left(\begin{array}{ccccc} 1 & \cdots & 0 & \cdots & 0\\ 0 & \cdots & 0 & \cdots & 0\\ 0 & \cdots & 1 & \cdots & 0\\ 0 & \cdots & 0 & \cdots & 0\\ 0 & \cdots & 0 & \cdots & 0 \end{array}\right)}}_{N_{U}\times N_{T}} \end{array}$$

if element 1 of row *i* is located in column *j*, it means that the *j*-th target is assigned to the *i*-th UAV. Thus, the target assignment can be achieved according to the *Q*-value ${\tilde{Q}}_{i,j}$ of each step, and the result of Hungarian transformation is used as the training label of the target assignment network.

(3) Construction of environmental state information with a new sequence

After the target assignment network of UAV *i* has been fully trained, a list of probabilities of UAV *i* moving to each target in the current state can be obtained, among which the assigned target has the largest probability in the list. As shown in the top section of the Figure 6, if the target *T*_*j*_ is assigned to UAV *i*, then the index of target *T*_*j*_ can be calculated by Equation (19),


(19) $$\begin{array}{l} index\left(T_{j}\right)=\text{argmax}\left(P_{i1},P_{i2},\ldots ,P_{iN_{T}}\right) \end{array}$$

The original environmental state information *o*_*i*_ = (*s*_*T*_1__, *s*_*T*_2__, ⋯ , *s*_*T*__*N*__*T*___, *s*_*U*_, *s*_*O*_) can be transformed into the environmental state information with new targets sequence õ_*i*_ = (*s*_*T*_*j*__, *s*_*T*_1__, ⋯ , *s*_*T*_*j*−1__, *s*_*T*_*j*+1__, ⋯ , *s*_*T*__*N*__*T*___, *s*_*U*_, *s*_*O*_), that is the assigned target *T*_*j*_ is placed in the first place of the target sequence to guide UAV *i* to recognize its own target.

The target assignment network realizes the optimal target assignment every step in the dynamic environment, and then UAV *i* uses the TD3 algorithm to plan the path for the assigned target according to the new state information ${\tilde{s}}_{i}=\left(s_{ui},{õ}_{i}\right)$. The Actor network updates according to the ${\tilde{Q}}_{i,n}$ and the state information ${\tilde{s}}_{i}$ with new sequence using Equation (20),


(20) $$\begin{array}{l} \nabla_{\phi_{i}}J\left(\phi_{i}\right)=N^{-1}\sum \nabla_{a}{\tilde{Q}}_{\theta_{i,1}}\left({\tilde{s}}_{i},a_{i}\right){|}_{a=\pi_{\phi_{i}\left({\tilde{s}}_{i}\right)}}\nabla_{\phi_{i}}\pi_{\phi_{i}}\left({\tilde{s}}_{i}\right) \end{array}$$

the TANet-TD3 is described in Algorithm 1.

**Algorithm 1:**
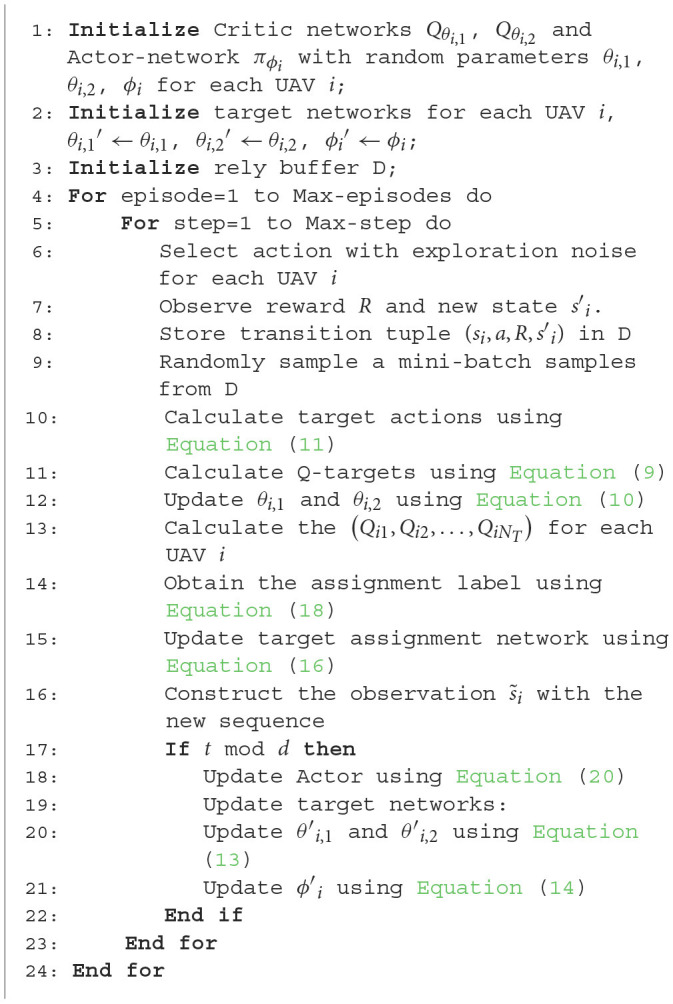
TANet-TD3

## 5 Experiments and results

In this section, the simulation environment is introduced first. Then, the training experiments, testing experiments, and statistical experiments are presented to verify the effectiveness of the proposed method in different scenarios.

### 5.1 Experimental settings

**Figure 7:**
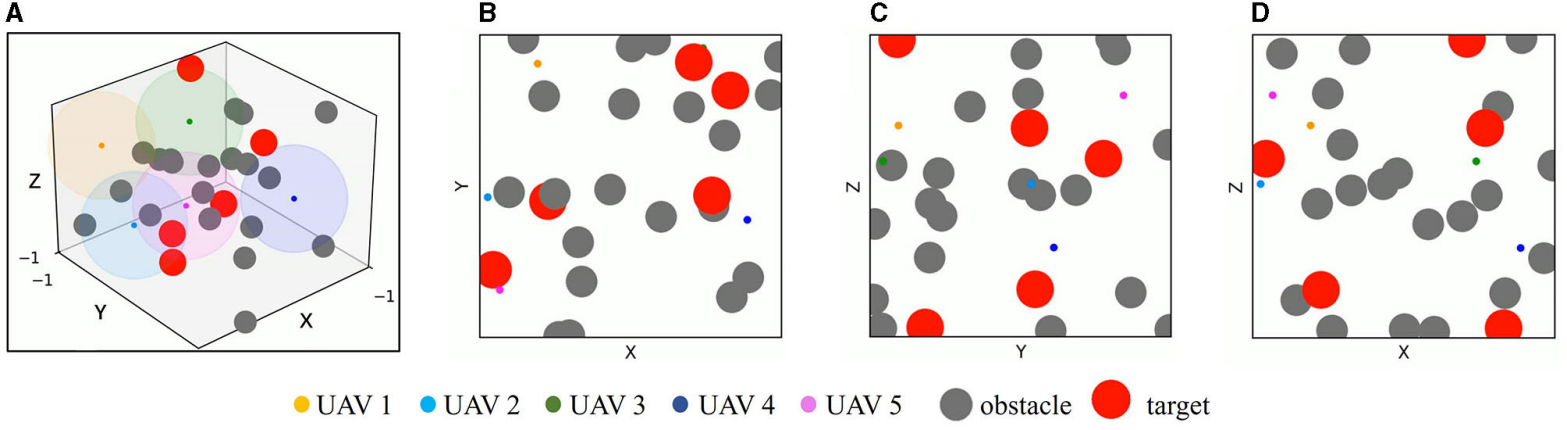
Simulation environment. **(A)** The 3D simulation environment. **(B)** The simulation environment from *X*–*Y* view. **(C)** The simulation environment from *Y*–*Z* view. **(D)** The simulation environment from *X*–*Z* view. The color spherical shade around UAV in **(A)** denotes the detection range of UAV.

A 3D simulation environment with two-dimensional three views is constructed based on the OpenAI platform to implement multi-UAV simultaneous target assignment and path planning in dynamic multiple obstacle environments. As shown in Figure 7, the simulation environment covers a 2 × 2 × 2 cubic area, UAVs, targets, and obstacles are simplified to a sphere and randomly initialized in this area. The radius of UAVs *r*_*u*_ = 0.02, and the maximum detection range *d*_det_ of UAVs is set as 0.5, which is denoted by the color spherical shades around UAVs. The radius of targets is set to *r*_*t*_ = 0.12. The obstacles have static mode and mobile mode with a radius *r*_*o*_ = 0.1, In motion mode, they move in a linear motion with a randomly initialized direction and velocity *v*_*i*_∈[−0.05, 0.05], *i*∈[*X, Y, Z*]. *v*_*i*_ represents the sub-velocity of obstacles in the *X, Y, Z* three directions. When it hits the boundary of the simulation environment, it moves in the opposite direction with the same velocity.

**Figure 8:**
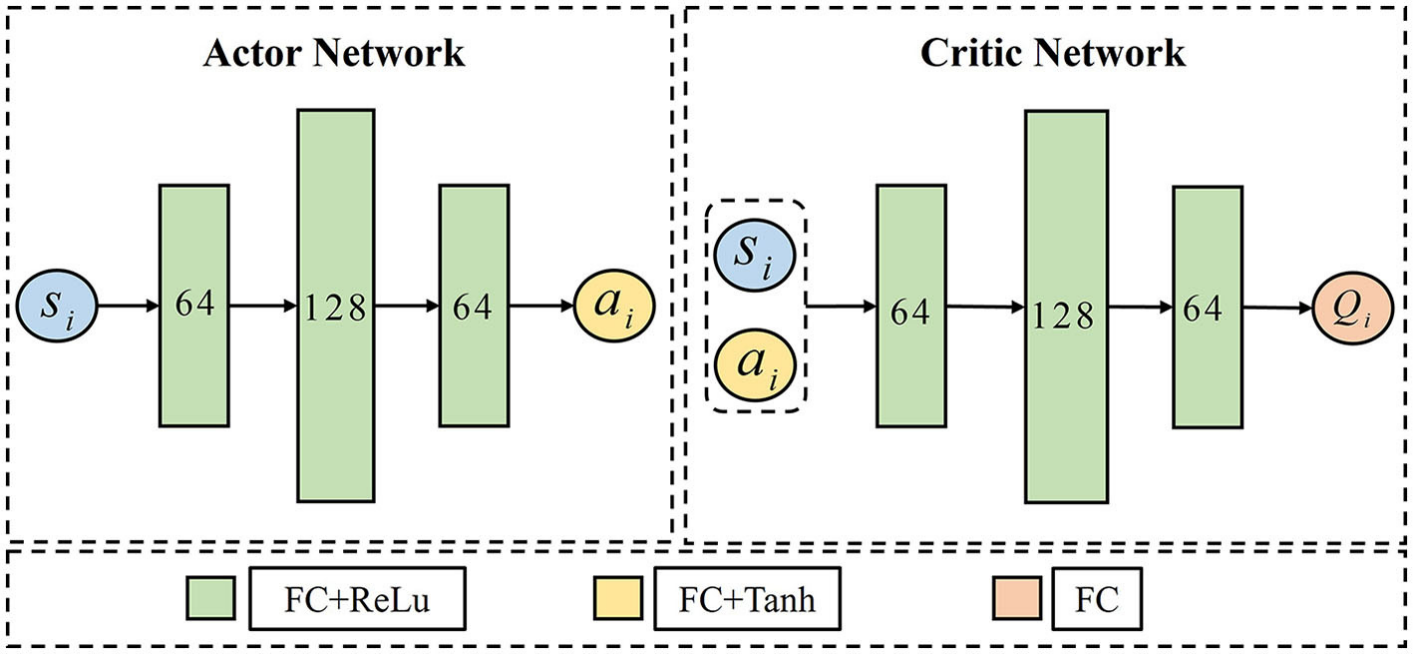
The network architecture of Actor and Critic in TD3.

In this paper, the network of TD3 is shown in Figure 8, *N* UAVs include *N* Actor-Critic structures. For UAV *i*, the Actor network is constructed by *s*_*i*_×64 × 128 × 64 × *a*_*i*_, where the input *s*_*i*_ represents the state of UAV *i*, and the output *a*_*i*_ represents the action performed by UAV *i*. The first three layers use a rectified linear unit (Relu) as the activation function, and the last layer uses a hyperbolic tangent (tanh) activation function to limit the output of action within the range of [−1,1]. The Critic owns a network structure of (*s*_*i*_+*a*_*i*_) × 64 × 128 × 64 × *Q*_*i*_, after three fully connected neural network layers (FCs) activated by Relu, the Critic maps the combination of state and action of UAV *i* to the *Q*-value evaluated by UAV *i*. The hyperparameters of TANet-TD3 and TD3 are given in Table 1.

**Table 1:** The hyperparameters of TANet-TD3.

**No**	**Hyperparameters**	**Values**
1	Max episodes number of TD3	5,000
2	Max episodes number of TANet-TD3	10,000
3	Max episodes length	100
4	Discount factor	0.9
5	Critic learning rate	1E-3
6	Actor learning rate	1E-4
7	Reply buffer size	5E5
8	Batch size	256
9	Soft update factor	0.01

### 5.2 Training experiments

Training experiments include two sections, the first section is to verify the advantages of TD3 in path planning, and the second section is to validate the effectiveness of TANet-TD3 in multi-UAV simultaneous target assignment and path planning. These algorithms have been trained in dynamic and mixed task environments as depicted in Figure 7, and in each episode, UAVs, targets, and obstacles are randomly initialized in the task area. There are three indicators used to measure the performance of training shown in Equation (21), including the average reward, the average arrival rate and the average target completion rate, where *N*_*ver*_ is the number of verification episodes, *R*_*i*-th_ is the reward of the *i*-th verification episode, $N_{i}^{U}$ is the number of UAVs that reach the target in the *i*-th verification episode and $N_{i}^{T}$ is the number of targets that have UAV reached in the *i*-th verification episode.


(21) $$\left\{ \begin{array}{l} \text{Average reward = }\sum_{i=1}^{N_{ver}}R_{i-\text{th}}/N_{ver}\\ \text{Average arrival rate = }\sum_{i=1}^{N_{ver}}N_{i}^{U}/(N_{ver}\times N_{U})\\ \text{Average target completion rate }=\sum_{i=1}^{N_{ver}}N_{i}^{T}/(N_{ver}\times N_{T})\; \end{array}\right.$$

#### 5.2.1 Training experiments for path planning tasks

Firstly, the TD3 algorithm is trained in single-UAV and multi-UAV dynamic scenarios respectively. Scenario I: one UAV, one target, and 20 moving obstacles; Scenario II: three UAVs, one target, and 20 moving obstacles. Each experiment is trained for 5,000 episodes, and 50 episodes of verification are conducted after every 10 episodes of training. The average reward and average arrival rate of these 50 verification episodes are counted to evaluate the algorithm. As a comparison, the DDPG algorithm is trained in the same task scenarios as TD3 with the same hyperparameters in Table 1.

**Figure 9:**
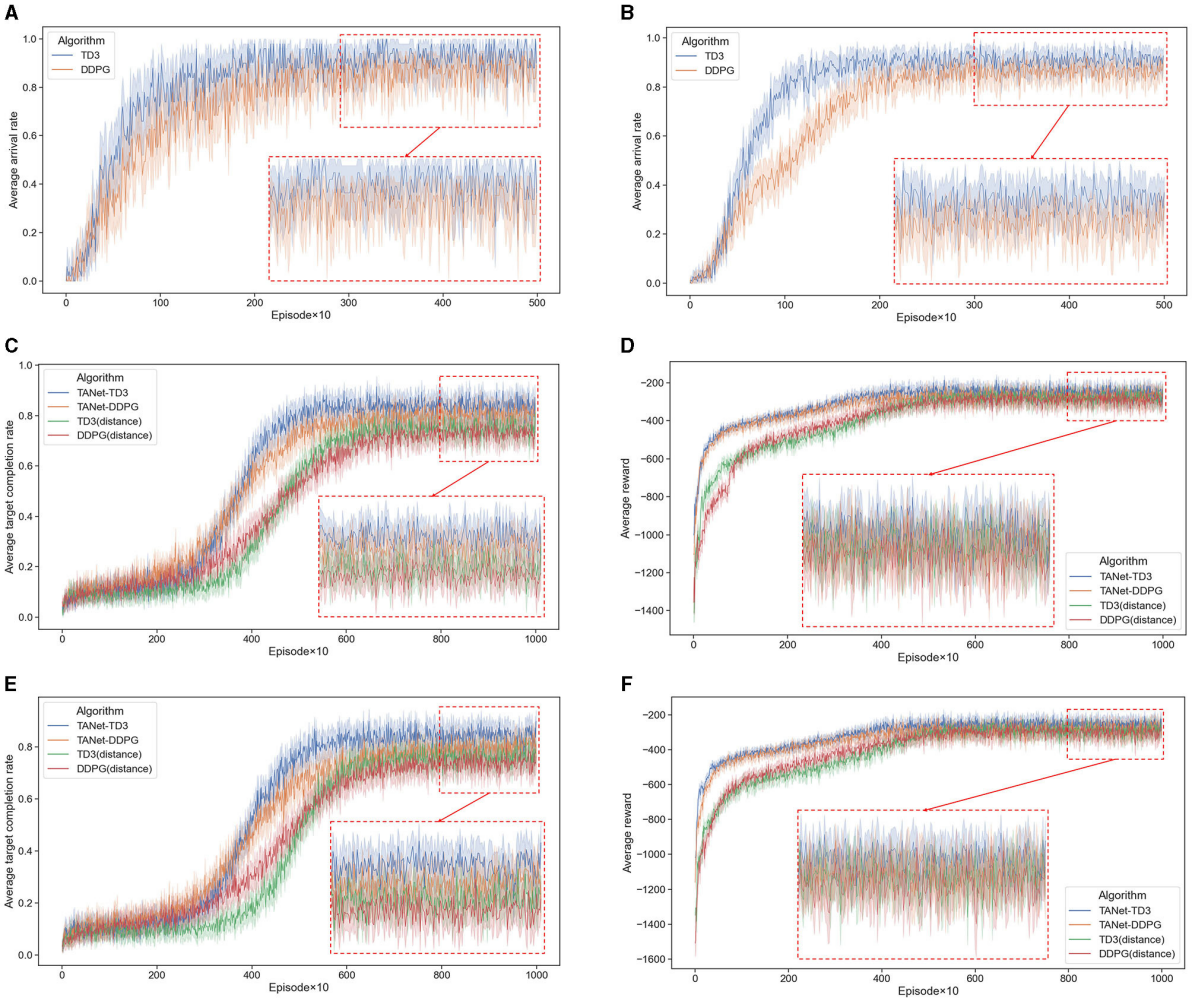
Convergence curves. **(A)** The average arrival rate of DDPG and TD3 in scenario I. **(B)** The average arrival rate of DDPG and TD3 in scenario II. **(C)** The average target completion rate of TANet-DDPG and TANet-TD3 in dynamic environment. **(D)** The average reward of TANet-DDPG and TANet-TD3 in dynamic environment. **(E)** The average target completion rate of TANet-DDPG and TANet-TD3 in mixed environment. **(F)** The average reward of TANet-DDPG and TANet-TD3 in mixed environment. The solid line denotes the statistical means and the 95% confidence interval of the means is shown shaded.

As can be seen from the training results depicted in Figures 9A, B, after adequate training, the TD3 algorithm has a good average arrival rate for path planning tasks, whether the scenario I of a single UAV (95%) or the scenario II of multiple UAVs (90%). It is evident that compared to the DDPG algorithm, TD3 has a better convergence effect and a faster convergence speed.

Therefore, in this paper, the TD3 algorithm is used as the basic algorithm for path planning, which can provide accurate assignment labels for the training of the target assignment network.

#### 5.2.2 Training experiments for simultaneous target assignment and path planning tasks

Next, the proposed algorithm TANet-TD3 is trained in the dynamic environment (five UAVs, five targets, and 20 moving obstacles) and the mixed environment (five UAVs, five targets, 10 static obstacles, and 10 moving obstacles). Each experiment has 10,000 episodes, and 50 episodes of verification are conducted after every 10 episodes of training. To verify the feasibility of the assignment network of TANet-TD3, DDPG with the target assignment network (TANet-DDPG) is introduced for comparison. In addition, the scheme of target assignment based on the distance between the target and UAV is introduced to the DDPG (DDPG(distance)) and TD3 (TD3(distance)) respectively to verify the advantages of TANet-TD3. Four algorithms are trained with the same hyperparameters in Table 1, and the target completion rate and the average reward are used as indicators to evaluate the performance of algorithms.

As shown in Figures 9C, E, in the initial stage, all algorithms generated training samples by the interaction process between UAVs and the environment, and the training started when the number of samples reached the capacity of batch size. The reward is very low and UAVs do not know what the goal is before the first 3,000 episodes. With the gradual rise of the samples in the reply buffer, each UAV gradually began to learn more intelligent strategies and finally reached the convergence result. The training results are listed in Table 2.

**Table 2:** The training results of TANet-DDPG and TANet-TD3.

**Environment**	**Algorithm**	**In episode 5,000**		**Last 1,000 episodes**	
		**Average target completion rate**	**Average reward**	**Mean average target completion rate**	**Mean average reward**
Dynamic	TANet-DDPG	71.20%	−303.5	80.70%	−271.5
	TANet-TD3	**81.54%**	**−241.4**	**83.77%**	**−253.0**
	DDPG(distance)	51.07%	−301.0	73.06%	−290.4
	TD3(distance)	55.27%	−296.5	75.10%	−275.0
Mixed	TANet-DDPG	63.20%	−305.9	80.38%	−281.1
	TANet-TD3	**78.21%**	**−220.5**	**84.27%**	**−255.2**
	DDPG(distance)	52.38%	−300.3	73.78%	−299.2
	TD3(distance)	48.00%	−295.9	76.28%	−289.2

The bold values represents the training result of our algorithm, and it is optimal among the four algorithms.

Figures 9C, D present that TANet-TD3 has the fastest convergence rate in the dynamic environment, reaching convergence about the 5,000th episode; followed by TANet-DDPG, while the TD3(distance) and DDPG(distance) algorithms reach convergence at about the 7,000th episode. Similarly, Figures 9E, F depict that TANet-TD3 has about 500 accelerated convergence compared to TANet-DDPG and about 2,000 accelerated convergence compared to TD3 and DDPG. Additionally, the relevant statistics in Table 2 illustrate that TANet-TD3 has the highest average target completion rate and average reward in both the dynamic and mixed environments. Compared to TANet-DDPG, TANet-TD3 leads an increase of (3.07%, 18.5) in a dynamic environment and (3.89%, 25.9) in a mixed environment. It has the largest average target completion rate difference of 10.71% (dynamic environment) and 10.49% (mixed environment) among TANet-TD3 and DDPG (distance).

Overall, the improvement of TANet-TD3 and TANet-DDPG is remarkable compared to DDPG(distance) and TD3(distance), this demonstrated that it is effective for the assignment method proposed in the paper, which can achieve simultaneous target assignment and path planning. Moreover, the training results also illustrated that TANet-TD3 outperforms TANet-DDPG in terms of convergence effect and convergence speed, which is mainly due to the superiority of TD3 in completing path planning tasks, which provides better *Q*-value labels for the optimization of target assignment network.

### 5.3 Testing experiments and results

**Figure 10:**
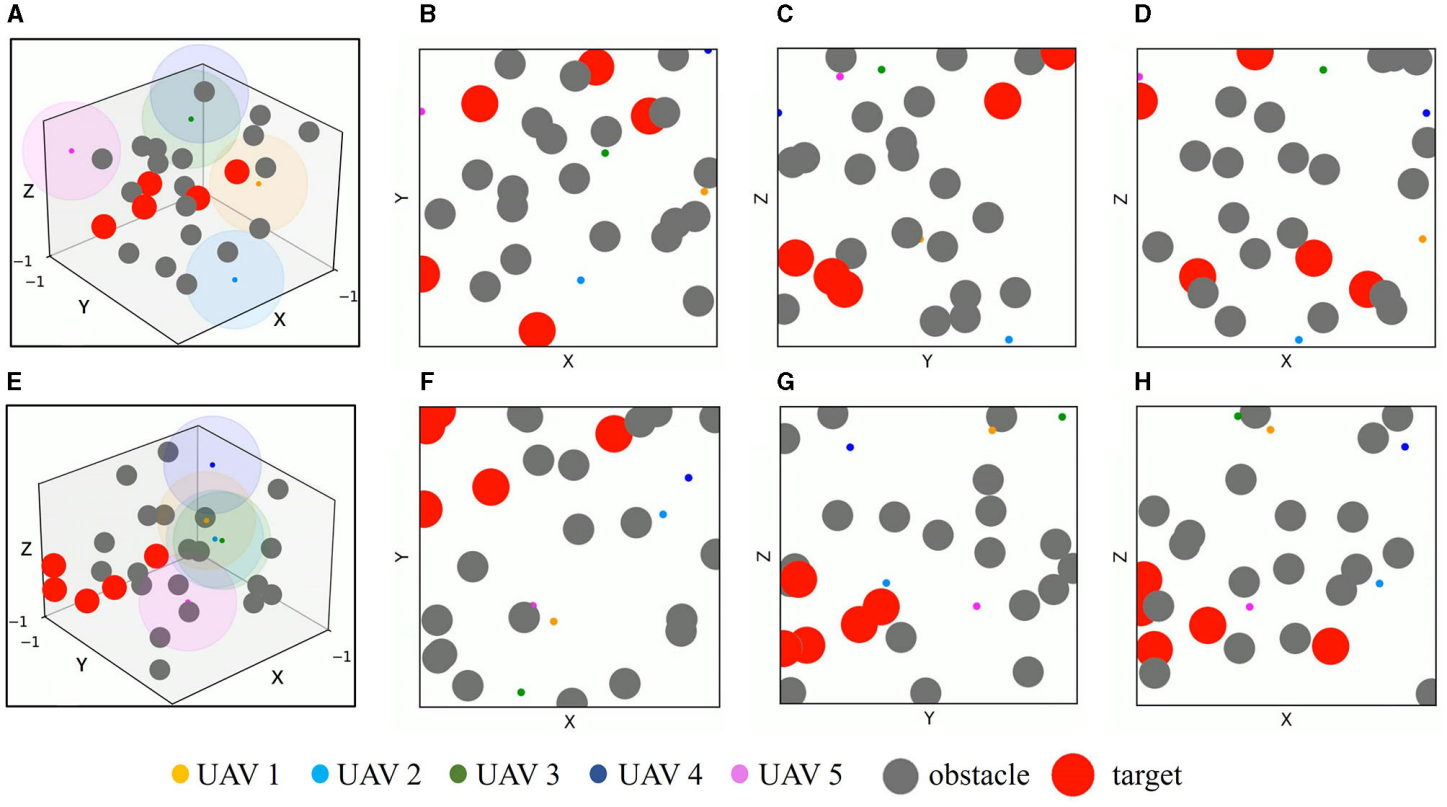
The test scenarios. **(A)** The 3D scenario of dynamic environment. **(B)** The dynamic environment from X-Y view. **(C)** The dynamic environment from Y-Z view. **(D)** The dynamic environment from X-Z view. **(E)** The 3D scenario of mixed environment. **(F)** The mixed environment from X-Y view. **(G)** The mixed environment from Y-Z view. **(H)** The mixed environment from X-Z view.

In order to evaluate the application efficiency of the algorithm after convergence and further verify the advantages of the TANet-TD3 algorithm in the simultaneous target assignment and path planning of multiple UAVs, a series of test experiments are conducted, in which the network parameters after convergence of TANet-TD3 and TANet-DDPG are used to control UAVs move in two environments. One is a dynamic environment, where all obstacles are mobile; the other environment is a mixed environment, where obstacles are static or mobile. As shown in Figure 10, UAVs, targets, and obstacles are randomly deployed in task areas, Figure 10A presents the 3D scenario of a dynamic environment with five UAVs, five targets, and 20 mobile obstacles, Figure 10B depicts the 3D scenario of the mixed environment with five UAVs, five targets, 10 static obstacles, and 10 mobile obstacles. Note that the colored spherical shades around UAVs represent the detection range of UAVs.

**Figure 11:**
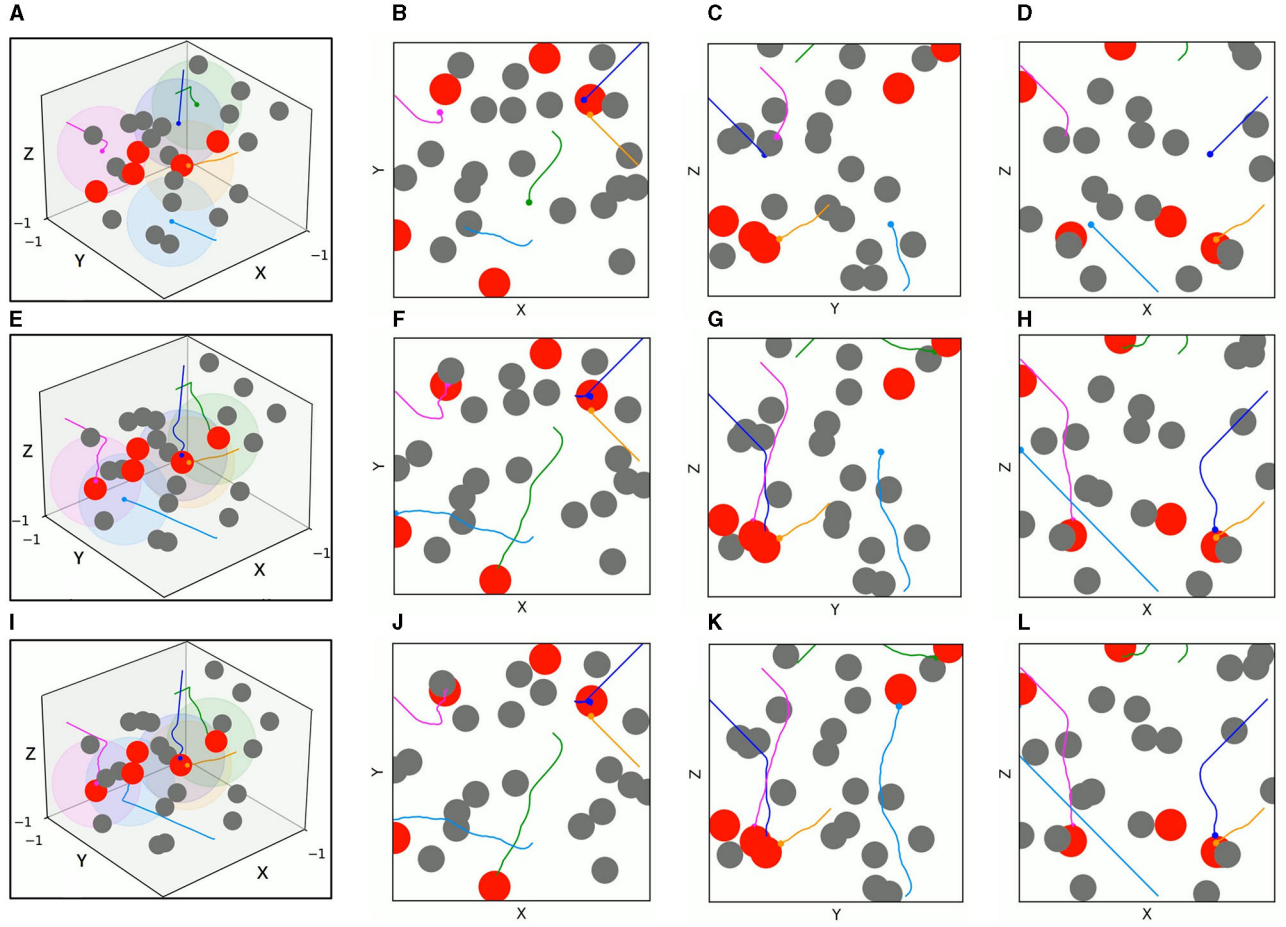
The 3D trajectories and corresponding 2D three views of UAVs driven by TANet-DDPG at different times in a dynamic environment. **(A)** The 3D trajectories at *t* = 4*s*. **(B–D)** The corresponding 2D three views of **(A)**. **(E)** The 3D trajectories at *t* = 8*s*. (**F–H)** The corresponding 2D three views of **(E)**. **(I)** The 3D trajectories at *t* = 12*s*. **(J–L)** The corresponding 2D three views of **(I)**.

**Figure 12:**
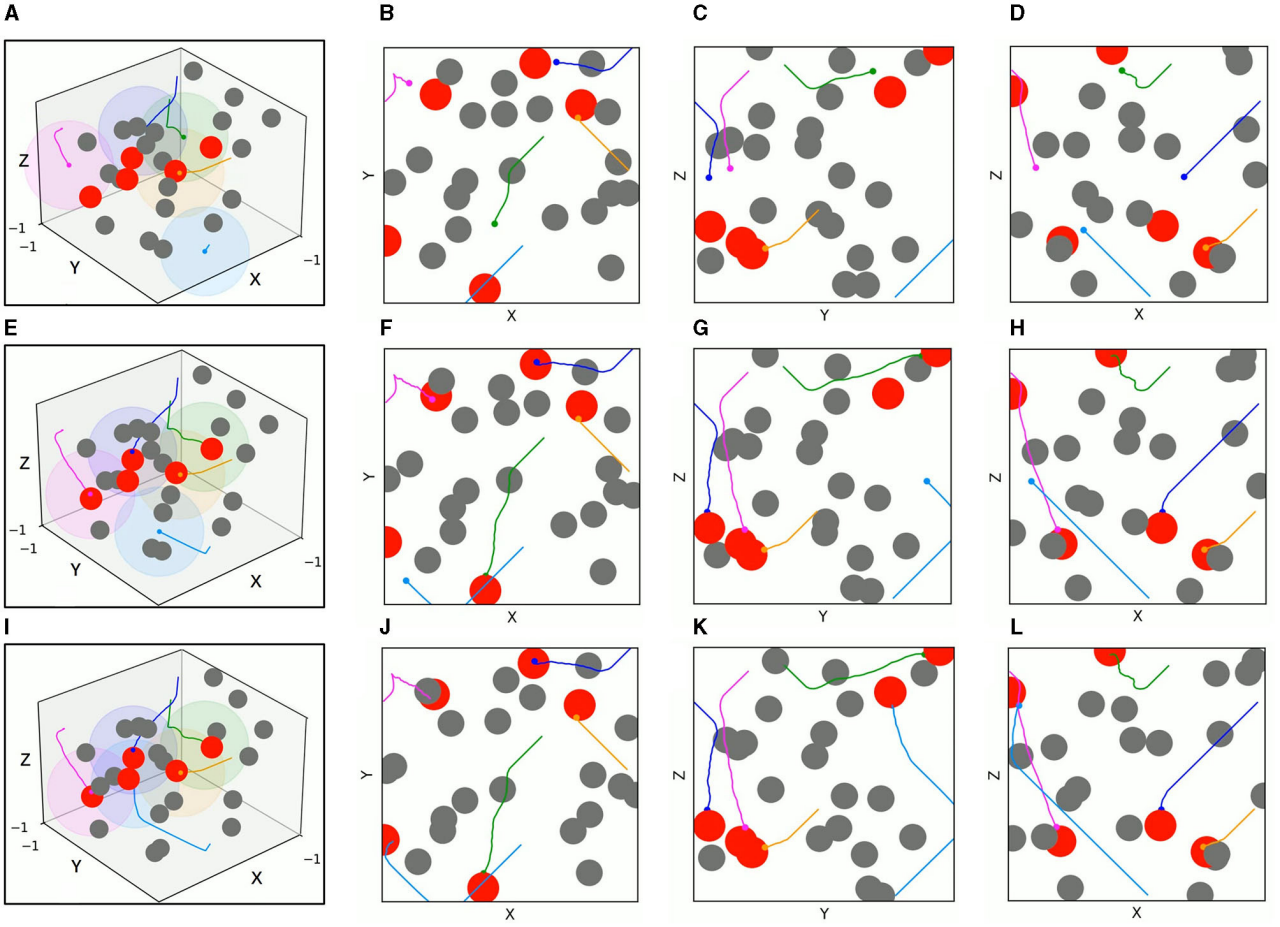
The 3D trajectories and corresponding 2D three views of UAVs driven by TANet-TD3 at different times in dynamic environment. **(A)** The 3D trajectories at *t* = 4*s*. **(B–D)** The corresponding 2D three views of **(A)**. **(E)** The 3D trajectories at *t* = 8*s*. **(F–H)** The corresponding 2D three views of **(E)**. **(I)** The 3D trajectories at *t* = 12*s*. **(J–L)** The corresponding 2D three views of **(I)**.

Figures 11, 12 present the 3D trajectories and corresponding 2D three views of five UAVs derived by TANet-DDPG and TANet-TD3 respectively in the dynamic scenario of Figure 10A. As can be seen in Figure 11, all five UAVs driven by TANet-DDPG reached targets, but UAV 1 and UAV 4 reached the same target resulting in one of five targets not having a UAV arriving for the next mission. Unlike TANet-DDPG, UAVs driven by TANet-TD3 achieve a full assignment of targets, that is a one-to-one correspondence between targets and UAVs. Meanwhile, it is evident that multiple UAVs execute simultaneous target assignment and path planning under TANet-TD3 and have a superior performance in the capability of obstacle avoidance. The trajectories of UAV 2 and UAV 5 in Figure 12 avoided the obstacle intentionally choosing a safer path as they approached the obstacle.

**Figure 13:**
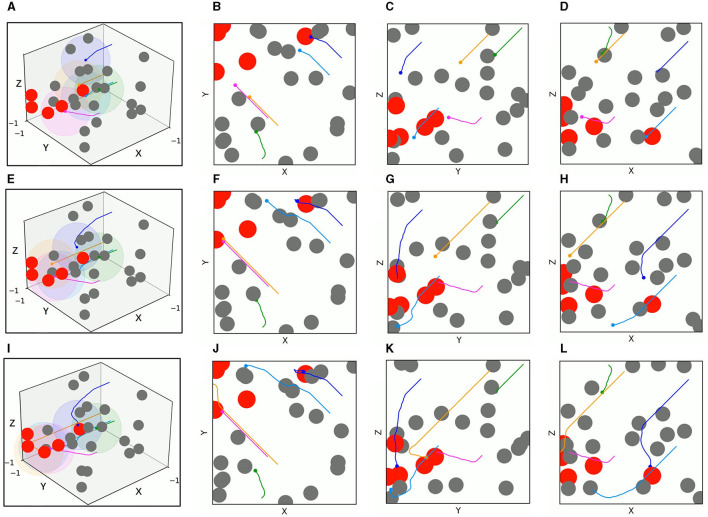
The 3D trajectories and corresponding 2D three views of UAVs driven by TANet-DDPG at different times in mixed environments. **(A)** The 3D trajectories at *t* = 4*s*. **(B–D**) The corresponding 2D three views of **(A)**. **(E)** The 3D trajectories at *t* = 8*s*. **(F–H)** The corresponding 2D three views of **(E)**. **(I)** The 3D trajectories at *t* = 12*s*. **(J–L)** The corresponding 2D three views of **(I)**.

**Figure 14:**
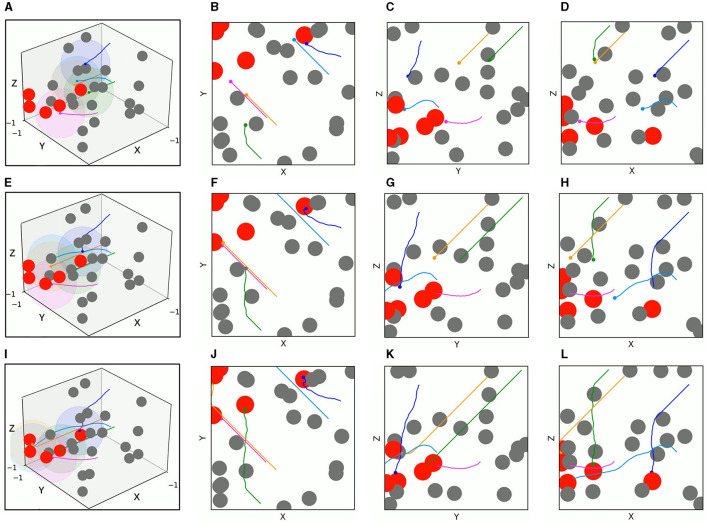
The 3D trajectories and corresponding 2D three views of UAVs driven by TANet-TD3 at different times in mixed environment. **(A)** The 3D trajectories at *t* = 4*s*. **(B–D)** The corresponding 2D three views of **(A)**. **(E)** The 3D trajectories at *t* = 8*s*. **(F–H)** The corresponding 2D three views of **(E)**. **(I)** The 3D trajectories at *t* = 12*s*. **(J–L)** The corresponding 2D three views of **(I)**.

The test results of the two algorithms in the mixed environment of Figure 10B are depicted in Figures 13, 14, respectively. First, UAV 2 and UAV 3 driven by TANet-DDPG failed to reach their assigned target due to hitting moving obstacles during flight, but they adapted well to the uncertain environment driven by TANet-TD3, and both succeeded in reaching their respective targets. Then, UAV 1 flew to the target reached by UAV 5 under the TANet-DDPG planning. In contrast, the test result derived by TANet-TD3 provides a complete assignment and a path without collision.

As a result, TANet-TD3 presents a better adaptability to dynamic environments compared to TANet-DDPG. Besides, the test statical results shown in Table 3 illustrate that TANet-TD3 exceeds TANet-DDPG in both the number of targets reached by UAVs and the reward value. According to the design of the reward function in Section 3.3, the value of reward also reflects the length of the flight path of UAVs, which also indicates that the path derived by TANet-TD3 is shorter than that derived by TANet-DDPG.

**Table 3:** The test statical results of TANet-DDPG and TANet-TD3.

**Environments**	**Algorithms**	**The number of targets reached by UAVs**	**Rewards**
Dynamic	TANet-DDPG	4	−256.87
	TANet-TD3	5	−141.97
Mixed	TANet-DDPG	2	−452.70
	TANet-TD3	5	−335.05

### 5.4 Statistical experiments

In this section, the statistical experiments about different numbers of UAVs and different numbers of obstacles are presented to further verify the advantage of TANet-TD3.

#### 5.4.1 Adaptability to different numbers of UAVs

**Figure 15:**
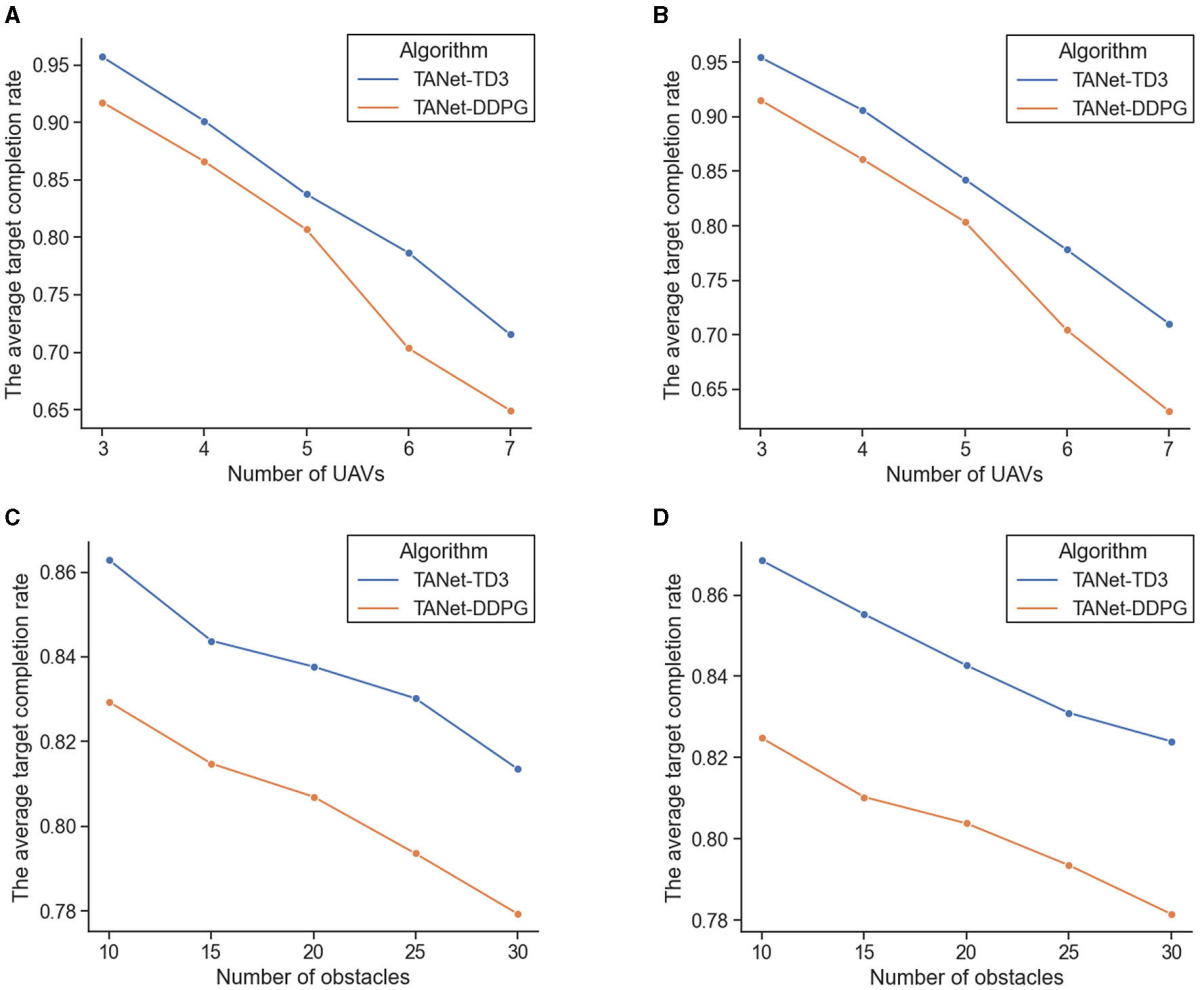
The comparison result of the average target completion rate of TANet-DDPG and TANet-TD3. **(A)** The comparison result under different numbers of UAVs in a dynamic environment. **(B)** The comparison result under different numbers of UAVs in mixed environments. **(C)** The comparison result under different numbers of obstacles in a dynamic environment. **(D)** The comparison result under different numbers of obstacles in a mixed environment.

In this experiment, the average target completion rate of the TANet-DDPG and TANet-TD3 are sequentially compared in terms of the number of UAVs from 3 to 7. The obstacles are set to 20 moving obstacles in a dynamic environment, and 10 static obstacles and 10 moving obstacles in a mixed environment. Each experiment with a specific number of UAVs is repeated 1,000 episodes, and in each episode, UAVs, targets, and obstacles are initialization with random position and velocity. Figures 15A, B depict the statistical results in dynamic environment and mixed environment, respectively.

As the number of UAVs increases, the difficulty of the simultaneous target assignment and path planning tasks increases dramatically, and the average target completion rate of TANet-DDPG and TANet-TD3 gradually decreases in both dynamic and mixed environments. Faced with the complex mission scenario of seven UAVs and 20 obstacles, TANet-TD3 can maintain an average target completion rate of more than 71% (71.54%, 71.06%). In contrast, TANet-DDPG has dropped to just over 70% (70.35%, 70.45%) at six UAVs and falls sharply below 65% (64.93%, 63.03%) at the seven UAVs. In addition, as the number of UAVs increases, the gap between TANet-DDPG and TANet-TD3 grows wider.

#### 5.4.2 Adaptability to different number of obstacles

This experiment verifies the effect of different numbers of obstacles on TANet-TD3 and TANet-DDPG. Specifically, the two algorithms are compared in dynamic and mixed environments with five UAVs and different numbers of obstacles including 10, 15, 20, 25, and 30, respectively. Each experiment is repeated 1,000 episodes, and the state of UAVs, targets, and obstacles are randomly initialized for each episode. The comparison results of the average target completion rate are presented in Figures 15C, D.

As shown in Figure 15, the increase in the number of obstacles has affected the performance of two algorithms both in dynamic and mixed environments, but TANet-TD3 consistently outperforms TANet-DDPG in all scenarios. Additionally, when the number of obstacles is 25, the average target completion rate of TANet-DDPG is below 80% in both dynamic (79.36%) and mixed environments (79.35%), while the average target completion rate of TANet-TD3 remains above 81% (81.36%, 82.40%) under the complex environment with 30 obstacles.

In summary, TANet-TD3 can effectively complete simultaneous target assignment and path planning. Besides, it has demonstrated that TANet-TD3 has a better adaptability to dynamic and random environments compared with TANet-DDPG.

## 6 Conclusion and discussion

This paper proposes a novel DRL-based method TANet-TD3 for multiple UAVs target assignment and path planning in dynamic multi-obstacle environments. The problem is formulated as a POMDP and a target assignment network is introduced to the TD3 algorithm to complete the target assignment and path planning simultaneously. Specifically, each UAV considers each target as its final target to be reached in turn and executes its action derived by TD3 for the next step. A *Q*-value matrix can be obtained by reward function and the Hungarian algorithm is used to act on the *Q*-value matrix to achieve an exact match between UAVs and targets. The matching result is used as labels to train the target assignment network, so as to obtain the optimal allocation for targets. Then each UAV moves to its assigned target under the planning of the TD3 algorithm. The experiment results demonstrate that TANet-TD3 can achieve simultaneous target assignment and path planning in dynamic multiple obstacle environments, and the performance of TANet-TD3 outperforms the existing methods in both convergence speed and target completion rate.

For future research, we will further improve the proposed method by combining it with specific applications, such as multi-UAV target search tasks and multi-UAV target-tracking tasks. Additionally, we will study the method of calculating the *Q*-value matrix in high-dimensional scenarios to deal with complex tasks with a large number of targets. Furthermore, we will build a more realistic simulation environment, in which the shape and movement of obstacles are more complex, to verify the effectiveness of the proposed algorithm.

## Data availability statement

The raw data supporting the conclusions of this article will be made available by the authors, without undue reservation.

## Author contributions

XK: Conceptualization, Methodology, Software, Validation, Visualization, Writing—original draft, Writing—review & editing. YZ: Supervision, Writing—review & editing. ZL: Supervision, Writing—review & editing. SW: Supervision, Writing—review & editing.
